# Effects of monitoring for visual events on distinct components of attention

**DOI:** 10.3389/fpsyg.2014.00930

**Published:** 2014-08-21

**Authors:** Christian H. Poth, Anders Petersen, Claus Bundesen, Werner X. Schneider

**Affiliations:** ^1^Neuro-Cognitive Psychology, Department of Psychology, Bielefeld UniversityBielefeld, Germany; ^2^Center of Excellence Cognitive Interaction Technology, Bielefeld UniversityBielefeld, Germany; ^3^Center for Visual Cognition, Department of Psychology, University of CopenhagenCopenhagen, Denmark

**Keywords:** visual attention, TVA, sustained attention, vigilance, salience, event-based prospective memory

## Abstract

Monitoring the environment for visual events while performing a concurrent task requires adjustment of visual processing priorities. By use of Bundesen’s (1990) Theory of Visual Attention, we investigated how monitoring for an object-based brief event affected distinct components of visual attention in a concurrent task. The perceptual salience of the event was varied. Monitoring reduced the processing speed in the concurrent task, and the reduction was stronger when the event was less salient. The monitoring task neither affected the temporal threshold of conscious perception nor the storage capacity of visual short-term memory nor the efficiency of top-down controlled attentional selection.

## INTRODUCTION

Monitoring the environment for visual events is a frequent requirement of everyday tasks. Monitoring errors can have tremendous consequences, for example when an anesthesiologist fails to detect aberrations in displays of a patient’s vital signs (see e.g., Seagull et al., 2001) or when an air traffic controller misses conflicting routes of air crafts (see e.g., Remington et al., 2000).

One may define *monitoring* as preparing for detecting visual events in the environment. Events could, for instance, refer to a change in appearance of a task-relevant object. In realistic situations, monitoring tasks can occur interwoven with a plethora of other activities. To date, however, little is known about the effects of monitoring for visual events on visual information processing within other concurrent activities.

For decades, monitoring has been investigated in the field of *vigilance* research (for an overview, see Davies and Parasuraman, 1982). Classical vigilance experiments required participants to detect and respond to rare visual events that occurred in between periods of visual stimulation without response requirements (e.g., Mackworth, 1948). In such tasks, detection performance typically declines over time. This result was termed “vigilance decrement,” and it has given rise to a conceptualization of vigilance as a “… psychological readiness to perceive and respond …” (Mackworth, 1948). Numerous explanations for the vigilance decrement have been proposed (Davies and Parasuraman, 1982) and are still debated (Hancock, 2013; see also Ariga and Lleras, 2011). The bulk of vigilance experiments comprised scenarios of preparatory monitoring during periods of low external stimulation where attention had to be sustained (Hancock, 2013). This research focused on the outcomes of monitoring, namely on detection performance. No studies asked how components of visual information processing are modified within the period of monitoring. In our investigation, we focus on the issue of how monitoring affects components of visual attention.

*Visual attention* summarizes a number of processes that govern priorities in visual information processing (e.g., Duncan, 2006; Chun et al., 2011). As a result of these priorities, currently relevant rather than irrelevant information is internally represented to be available for action control (Allport, 1987; Neumann, 1987; Desimone and Duncan, 1995; Schneider, 1995; Duncan, 2006). A framework for describing how this prioritization might be accomplished is provided by the “biased competition” approach. All inputs from the visual field may compete against each other for being available to control actions and this competition may be biased by representations of the current task (Desimone and Duncan, 1995). Such task representations define sets of processing priorities (“attentional sets”) and may stem from working memory (e.g., Olivers et al., 2011) or long-term memory (e.g., Woodman et al., 2013).

A specific biased competition theory about distinct attentional components was suggested by Bundesen’s (1990) computational *Theory of Visual Attention* (TVA). TVA assumes visual information processing to be organized in separate processing stages. Visual short-term memory (VSTM) is the stage at which visual information is temporarily maintained and becomes available for action control, so that it can be reported or acted upon. At a preceding stage, competition of objects in the visual field takes place. This competition is characterized as a race of possible categorizations of objects. The categorizations that finish processing first win the race and enter VSTM, provided there is still a free “slot” for storing information about the respective object (for evidence for such a capacity limitation of VSTM, see Luck and Vogel, 1997). In TVA, two attentional mechanisms (*filtering* and *pigeonholing*) jointly determine which of the categorizations of objects in the visual field win the race and are encoded into VSTM. TVA’s filtering mechanism was used to explain observations of “focused attention” where processing was concentrated on relevant at the expense of irrelevant visual objects (for a review, see Bundesen and Habekost, 2008). Visual processing appears to be limited in capacity so that only a certain number of visual objects can be processed at a given time (e.g., Duncan, 2006). Filtering is assumed to operate by assigning each object in the visual field an attentional priority reflecting its current relevance and salience (Nordfang et al., 2013). In TVA, these attentional priorities of objects are called “attentional weights.” The processing speed of a categorization of an object is proportional to the absolute attentional weight allocated to this object, normalized by the attentional weights allocated to all objects in the visual field. Therefore, filtering directly affects the probability that an object-categorization finishes processing earlier than others and wins the race. TVA’s second attentional mechanism, pigeonholing, consists of biases for categorizing visual objects as belonging to certain task-relevant categories. Like filtering, it is assumed to influence the processing speed of categorizations and thus their probability of winning the race to VSTM.

Using a TVA-based letter-report task (Vangkilde et al., 2011; see below), five distinct attentional components can be obtained. Each component represents a specific aspect of attentional functioning (for overviews, see also Bundesen and Habekost, 2008, 2014). (1) The threshold of conscious perception, *t*_0_, is the maximum ineffective exposure duration of a visual stimulus. That is, it indexes the time necessary to initiate the race of object categorizations to VSTM. (2) The maximum number of visual objects that can be maintained in VSTM, *K*, is taken as a measure of VSTM storage capacity. (3) Visual processing speed, *C*, reflects the overall available visual processing capacity which is distributed across the objects in the visual field according to their attentional weights. (4) The top-down controlled attentional selectivity, α, represents the efficiency of TVA’s filtering process. It captures how efficiently the processing of relevant visual objects can be prioritized over the processing of irrelevant ones. (5) The laterality of attentional weighting, *w*_index_, provides an index of spatial attentional biases that lead to preferred encoding of visual objects from one of the visual hemifields (cf. Duncan et al., 1999; Vangkilde et al., 2011). To date, these TVA-based components have been applied to characterize attentional performance in a variety of psychological, neuropsychological, and physiological studies (for overviews, see Bundesen and Habekost, 2008, 2014). They may thus provide suitable measures to capture effects of event monitoring on visual attention.

Whether or not monitoring affects visual attention components might be especially important in situations where, concurrently to monitoring, another task has to be performed. In the above sketched real-life situation, an anesthesiologist might have to prepare for detecting changes in visual displays of a patient’s vital signs while performing other tasks, such as the administration of anesthetics. Such situations may be reminiscent of tasks of event-based prospective memory. In such tasks, the intention to respond to an external event is formed and maintained over a retention interval while another ongoing task is performed, and then enacted when the event actually occurs (for reviews, see Ellis and Kvavilashvili, 2000; Burgess et al., 2011). During this retention interval, performance in the ongoing task seems impaired (e.g., Smith, 2003). In the prospective memory literature, this finding of task interference was interpreted as evidence for an overlap of attentional processing resources recruited by both, the ongoing and the event-based prospective memory task. Consequently, preparatory attentional processes were proposed, which monitor the environment for events to support event-based prospective memory (Smith, 2003; Smith and Bayen, 2004). It was implicitly assumed that only one form of attentional resource exists that has to be shared between tasks (for a critical discussion, see Allport, 1987; Neumann, 1987). In this view, tasks may either consume this resource or be “automatic” without resource consumption (for a critical discussion, see Neumann, 1984). Using TVA-based assessment, this suggestion of sharing one general attentional resource can be replaced by assuming several independent attentional resource components such as VSTM storage capacity, attentional weighting and top-down selectivity.

One factor that may be critical for effects of monitoring for visual events on visual attention may be the perceptual salience of the events. Salience plays a central role in all three of the mentioned research domains. Firstly, performance in detecting rare events seems not to decline with time-on-task when events are highly salient (Helton and Warm, 2008). Secondly, visual stimuli of high salience can capture visual attention, that is, can intrinsically call for a prioritization in processing over low-salience stimuli (e.g., Theeuwes, 2004). Thirdly, the attentional demands made by event-based prospective memory tasks are thought to be lower in case of highly salient events (e.g., McDaniel and Einstein, 2000; Hicks et al., 2005). Therefore, the effects of monitoring for visual events on visual attention could vary depending on the expected perceptual salience of the events. For instance, monitoring for highly salient events might rely more on the expected ability of the events to capture attention. This may lead to weaker effects on visual attention in a concurrent ongoing task, compared to monitoring for events of lower salience.

The aims of the present study were to investigate, first, how monitoring for visual events affects the distinct TVA-based attentional components in a concurrent ongoing task and, second, whether these effects vary with the expected salience of the events. The experiment consisted of two overlapping tasks. First, participants performed an ongoing letter-report task enabling the TVA-based estimation of the attentional components (cf. Bundesen, 1990; Vangkilde et al., 2011). Second, event monitoring was required while engaging in the ongoing letter-report task. Events were brief luminance increases of a central fixation cross. Each participant performed three conditions. In the *low-* and *high-salient event condition*, participants monitored for and responded to events. Events were of lower salience in the former than in the latter condition. In the *control condition*, events were as salient as in the low-salient event condition, but participants were not required to monitor for them or to respond to them. Importantly, we attempted to capture only effects on the attentional components that were due to the preparatory monitoring for events. Therefore, only trials containing neither events nor related responses were analyzed. Moreover, we aimed at keeping the extent to which events were monitored constant for each of the two event conditions by regularly giving feedback about detection performance. This feedback (cf. Ariga and Lleras, 2011) and possibly the structure of the letter-report task should have prevented time-on-task effects, such as vigilance decrements (McAvinue et al., 2012; see also Matthias et al., 2010). In addition to our first experiment (Experiment A), we conducted a replication experiment comprising identical experimental conditions (Experiment B).

How might event monitoring affect the TVA-based attentional components? First, monitoring for a visual event could involve the maintenance of a representation of this event in VSTM to enable its use to define the attentional set for the task (cf. Olivers et al., 2011). This requirement should have lowered the storage capacity available to maintaining letters of the letter-report task. In this case, the estimated VSTM capacity should have been lower in the two event conditions compared to the control condition where monitoring was not necessary. In addition, if the salience of events affected the need for such a representation, then the estimated VSTM capacity should also differ between the two event conditions.

Second, the event-based prospective memory literature suggests that monitoring should consume unspecific and capacity-limited attentional resources (Smith, 2003; Smith and Bayen, 2004). In the framework provided by TVA (Bundesen, 1990), specific attentional resources such as visual processing speed are assumed. In the present experiments, more visual processing resources should be assigned to process the fixation cross when it is monitored for events compared to when it is not. Under such circumstances, less resources should be available for processing the letters of the letter-report task. Therefore, the processing speed of the letters should be reduced in the event conditions compared to the control condition. Moreover, events with a higher salience could intrinsically call for visual processing resources (e.g., Theeuwes, 2004) which might lessen the amount of resources that must be reserved for monitoring potential events. In the high-salient event condition, this should lead to more available resources for processing the letters and thus to a higher processing speed compared to the low-salient event condition.

In addition to the TVA-based components of VSTM capacity and visual processing speed, we explored potential effects of monitoring on the threshold of conscious perception, the top-down controlled selectivity, and the laterality of attentional weighting and examined the potential role of the salience of events.

To preview the results, the monitoring manipulation affected the processing speed as predicted and, additionally, the laterality of attentional weighting. However, no effects of monitoring were observed with respect to VSTM capacity, the threshold of conscious perception, and the top-down controlled attentional selectivity.

## MATERIALS AND METHODS

### PARTICIPANTS

Twenty students (13 females, 7 males) from the University of Copenhagen, Denmark took part in Experiment A, receiving a shopping voucher for participation. Four participants stated being left-handed, 16 being right-handed. Their ages ranged from 20.28 to 34.26 years, with a mean age of 24.92 (SD = 2.91).

Nineteen students (13 females, 6 males) from Bielefeld University, Germany, participated in Experiment B, some of whom received course credit for participation. Three reported being left-handed, 16 right-handed. The participants were between 19.91 and 31.75 years old, with a mean age of 25.91 (SD = 3.15).

All participants of both experiments reported having normal or corrected-to-normal visual acuity as well as normal color vision. Written informed consent was obtained from all of them before participation.

### APPARATUS AND STIMULI

Participants performed Experiment A in a dimly lit room at the University of Copenhagen, wearing disconnected headphones (SRH 440, Shure, Niles, IL, USA) to be shielded from sounds. Stimuli were displayed on a 21″ or a 19″ CRT-monitor (G220f, ViewSonic, Walnut, CA, USA, or Flatron 915 FT plus, LG, Seoul, South Korea, respectively) running at 100 Hz with a resolution of 1024 × 768 pixels corresponding to physical dimensions of 40 (width) × 30 (height) cm or 36 (width) × 27 (height) cm, respectively. One participant performed the complete experiment with a screen refresh rate of 120 Hz. The participant’s data was analyzed because the applied modeling procedure took the refresh rate into account, so that estimations of the attentional components should not have been affected. The experiments were programmed and conducted using the E-Prime 2.0 software, ensuring accurate timing of the visual presentation (see Schneider et al., 2002). Participants viewed the screen from a distance of approximately 60 cm. Responses were recorded using a standard computer keyboard.

Stimuli were presented on a black background. A gray “plus”-character written in Bell MT font with a size of 18 pt., corresponding to approximately 0.5° × 0.5° of visual angle, and with a luminance of 33.12 cd/m^2^ was used as central fixation cross. Letter stimuli consisted in red (RBG: 253, 43, 43; 24.57 cd/m^2^) and blue (RBG: 43, 53, 255; 14.05 cd/m^2^) capital letters, written in Arial font with a size of 68 pt., corresponding to approximately 2.7° × 2.3° of visual angle. Letter masks were made of red and blue letter fragments and covered an area of 100 × 100 pixels. Events were luminance increases of the fixation cross to either 43.20 cd/m^2^ in the low-salient event and control conditions or to 111.57 cd/m^2^ in the high-salient event condition.

Experiment B took place in a dimly lit and sound-attenuated room at Bielefeld University. Stimuli were shown on a 19″ CRT-monitor (G90fB, ViewSonic, Walnut, CA, USA) with a resolution of 1024 × 768 pixels, corresponding to physical dimensions of 36 (width) × 27 (height) cm. Participants viewed the screen from a distance of 71 cm, while their head was stabilized by a chin-rest. The stimuli of Experiment B were identical to those of Experiment A, except for size and luminance. The dimensions of the central fixation cross were 0.4° × 0.4° of visual angle and its luminance was 42.00 cd/m^2^. Letter stimuli covered 2.3° × 1.9° of visual angle. The luminance of the red target letters was 26.00 cd/m^2^, the one of the blue distractor letters 18.00 cd/m^2^. Events were luminance increases of the fixation cross to 51.00 cd/m^2^ in the low-salient event and control conditions and to 85.00 cd/m^2^ in the high-salient event condition.

### PROCEDURE AND DESIGN

**FIGURE 1:**
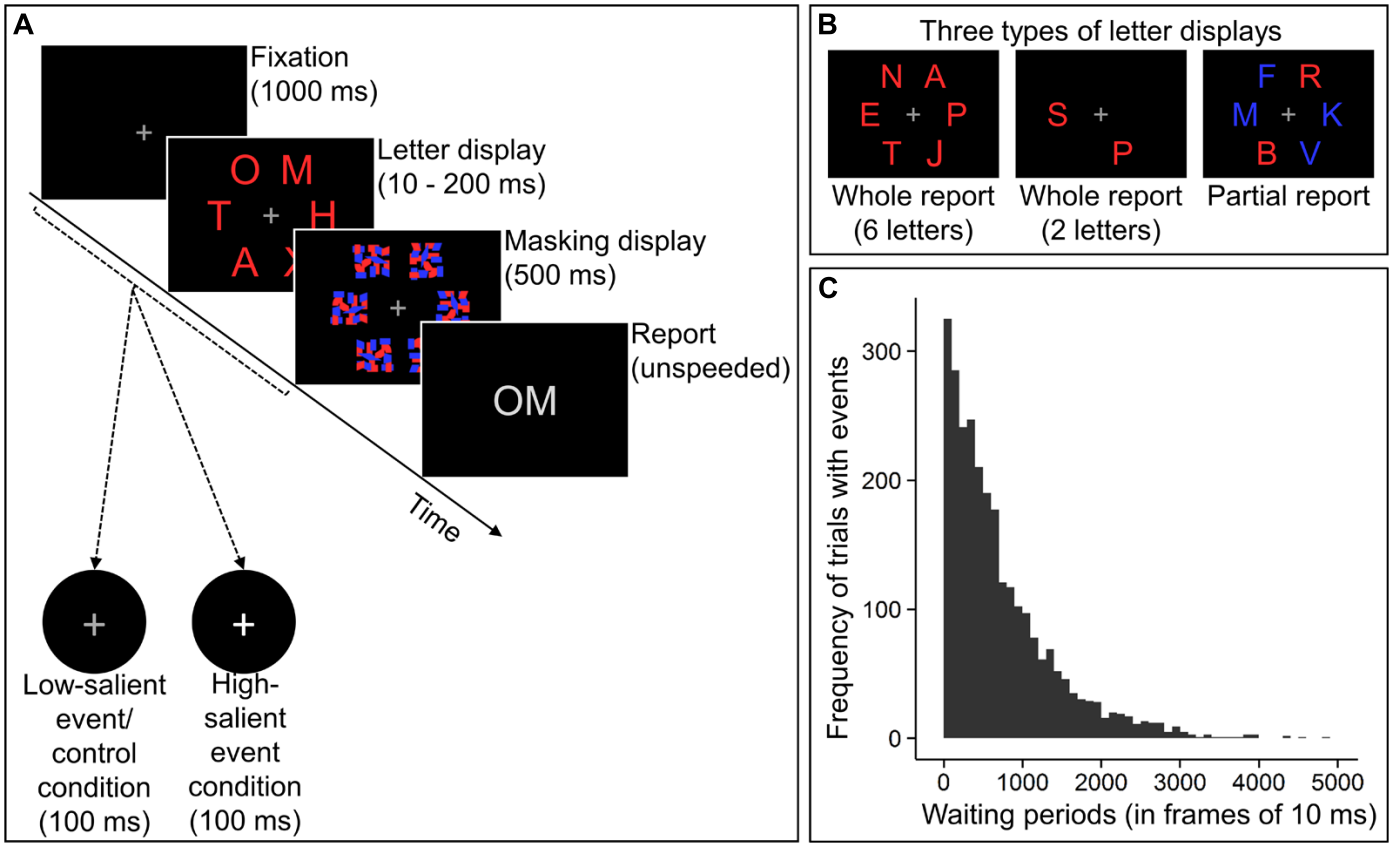
**Experimental paradigm. (A)** Course of an example trial. Participants viewed a fixation cross, followed by a letter display which was masked afterward. Then they were to report the presented letters. On 10% of all trials, events consisting in brief luminance increases of the fixation cross occurred. Participants had to respond to luminance increases in the low-salient event and high-salient event condition, but not in the control condition. Luminance increases were greater in the high-salient event compared to the other two conditions. **(B)** Letter displays could be of three different types, enabling the estimation of attentional components (Vangkilde et al., 2011). **(C)** Across each condition, waiting periods to events approximated the non-aging geometric distribution.

The experimental task for measuring the TVA-based attentional components consisted of a modified version of the CombiTVA-paradigm, a letter-report task combining whole and partial report techniques (Vangkilde et al., 2011). **Figure 1A** illustrates the course of an example trial. Each trial began with the presentation of a central fixation cross for 1000 ms. Then a letter display was presented that could be one of three types (see **Figure 1B**). In whole report trials, either two or six red target letters were presented whereas partial report trials comprised two red target letters and four blue distractor letters. In each block, nine 2-letter and eighteen 6-letter whole report as well as nine partial report trials were administered in mixed order. Letters in each trial were chosen randomly without replacement from the set of all capital letters except I, Q, U, and W, which were omitted to reduce confusability between letters. Letter displays containing six target letters were presented for either 10, 20, 50, 80, 140, or 200 ms. All other letter displays were shown for 80 ms. Letters were presented at 45, 90, 135, 225, 270, and 315° of an imaginary circle with a radius of approximately 7.5° of visual angle (6.35° in Experiment B) around the fixation cross. The letter display was followed by a masking display shown for 500 ms, which always contained six letter masks at the six possible letter locations. Then a response screen was presented on which participants could enter letters using the keyboard. These letters were displayed until participants pressed the “enter”-key to start the next trial. The participants’ task was to report only the red target letters, while ignoring the blue distractor letters. They were instructed to type in the letters (in arbitrary order) they were “fairly certain” of having seen, refraining from pure guessing. Moreover, they were instructed to aim at an accuracy of their letter reports between 80 and 90% (i.e., at error rates between 10 and 20%). The participant’s accuracy level was displayed after each block.

In 10% of all trials, an event (i.e., luminance increase of the fixation cross) was presented for 100 ms. The event’s onset could occur either during the sole presentation of the fixation cross, during the letter display, or during the first 400 ms of the masking display (i.e., the first, second, and third displays in **Figure 1A**). The following steps were taken to keep the temporal expectation of events constant throughout each condition of the experiment. The specific trials on which an event occurred in each condition were randomly determined for each participant. For these trials, the onset of the event was set to a frame in the refresh cycle of the screen that was randomly chosen as well. Only one event could appear in each of those trials. Simulations of 75 complete runs of one condition revealed that the waiting periods (measured in frames of 10 ms) to the occurrences of the first, second, etc., and last event each approximated geometric distributions as assessed by diagnostic histograms. Waiting periods across all occurrences of the events approximated a geometric distribution with a probability parameter of 0.00138401 (maximum-likelihood), corresponding to a mean waiting period of 7.23 s (723 frames of 10 ms). A histogram of these overall waiting periods is depicted in **Figure 1C**. Waiting times following non-aging probability distributions have been used previously to establish constant temporal expectations (Vangkilde et al., 2012).

The monitoring task was manipulated as an independent variable in a within-subject design. Each participant performed the three different conditions, each comprising nine blocks of 36 trials, and 36 additional trials that were distributed across the nine blocks and each contained an event. In the low-salient event and high-salient event conditions, participants were required to press the space-bar as quickly as possible when an event was presented. Participants were told that it was very important not to overlook the events and they were instructed to refrain from reporting letters on these trials. The two event conditions differed only insofar as events in the high-salient event condition were greater luminance increases than in the low-salient event condition. After each block in these conditions, the number of events the participants correctly responded to and the number of missed events were displayed on the screen. In the control condition of the monitoring task, luminance increases of the fixation cross were presented that were identical to those of the low-salient event condition, but participants were instructed not to respond. They were, however, informed that irrelevant luminance increases of the fixation cross occurred.

Experiments A and B both took place in two sessions of approximately 90 min, on two days. In Experiment A, participants were examined either individually or in groups of up to five participants, separated by non-transparent curtains. In Experiment B, participants always performed the experiment alone. Each session comprised all three conditions. Prior to each condition, participants performed one block of training trials. The order in which the conditions were administered in the first session was reversed in the second, to control for effects of fatigue. The overall order of conditions was counterbalanced across the sample. Prior to the start of each condition, participants read written instructions on the screen and reported them to the experimenter in their own words. In case participants did not report the instructions correctly, the experimenter repeated the instructions verbally after which participants paraphrased them again.

### DEPENDENT MEASURES

Measures of monitoring performance were based on the hits (i.e., space-bar presses following events within the same trial) and false alarms (i.e., space-bar presses on trials without events). The non-parametric measures of signal detection, A′ and $B_{D}^{\prime \prime }$ (Donaldson, 1992) were used to quantify the participant’s sensitivity in detecting events and their bias for reporting an event, respectively. Values for A′ range from 0.50 (chance level) to 1.00 (perfect sensitivity). Values for $B_{D}^{\prime \prime }$ range from -1.00 to 1.00. Negative values indicate a bias to respond that an event had been presented, a value of 0.00 indicates no bias, and positive values indicate a bias to respond that no event had been presented (Donaldson, 1992). In addition, reaction times (RTs; in ms) for correct responses to events were collected.

Because participants were instructed to aim at an accuracy level of their letter reports between 80 and 90%, their error rates in the three conditions (the proportion of typed-in letters that were wrong) were collected as a control variable. The five TVA-based attentional components (see below) were estimated for each participant in each of the three conditions. These estimates were obtained using a maximum-likelihood fitting procedure based on the number of correctly reported target letters for the different trial types (i.e., 2 and 6-letter whole report and partial report trials) and presentation durations of the letter displays (an overview of how the attentional components are estimated from performance in the CombiTVA-paradigm is given by Vangkilde et al., 2011; for a detailed description of the fitting procedure, see Kyllingsbæk, 2006 and Dyrholm et al., 2011). This means that these estimations only took the letters of the letter-report task into account. Since performance in event detection was assessed separately, the fixation cross was not included in estimations of the attentional components. A model with 14 free parameters was fitted using the LIBTVA-toolbox (Dyrholm et al., 2011; see also Habekost et al., 2014) for MATLAB^®^ (R2011a, The Mathworks, Natick, MA, USA). The first three attentional components were estimated from the numbers of correctly reported target letters on 6-letter whole report trials.

(1) The threshold of conscious perception, *t*_0_, is defined as the lowest presentation duration (measured in ms) required to initiate a race between categorizations for encoding into VSTM. It is assumed to be drawn from a normal distribution with a given mean and standard deviation (two free parameters) on a trial-by-trial basis.
(2) The storage capacity of VSTM, *K* (measured by the number of retained letters), is assumed to be drawn from a probability distribution on each trial that has five free parameters, namely the probability that *K* takes a value of 1, 2, …, 5. The probability that *K* is 6 equals 1 minus the sum of the five parameters. The final estimate of *K* consists in the value that is expected on the basis of its probability distribution.
(3) The total visual processing capacity or visual processing speed, *C* (measured in letters/s), is estimated as follows. In the traditional TVA model, the categorizations of the letters are thought to be processed in parallel once *t*_0_ has passed whereby their processing times are assumed each to follow an exponential distribution. The rate parameters of these distributions reflect the speed at which the letter categorizations are processed. The processing speed, *C*, is the sum of these rate parameters and it is thought to be a constant (one free parameter). For the current model, the assumption of normally distributed values for *t*_0_ implied an ex-Gaussian distribution of the processing times (Dyrholm et al., 2011). The last two attentional components are based on the estimation of attentional weights for target letters at each of the six locations (five free parameters).
(4) The top-down controlled attentional selectivity is captured by comparing the participants’ performance on partial report trials with their performance on 2-letter whole report trials. If the four distractor letters on partial report trials do not exert any distracting effects on performance, then participants should perform equally in reporting the two target letters on partial report and on 2-letter whole report trials. The top-down controlled attentional selectivity is quantified by the free parameter α, the attentional weight of a distractor letter relative to the attentional weight of a target letter. An efficient selection leads to α values close to 0, an inefficient selection with no prioritization of target letters over distractor letters, to α values close to 1.
(5) The fifth attentional component, *w*_index_, represents the laterality of attentional weighting across both visual hemifields, assessed as the sum of the attentional weights allocated to letters in the left visual hemifield in relation to the sum of all attentional weights in the entire visual field. Thus, *w*_index_ values of 0.5 indicate no spatial bias, whereas values close to 0 show a right-sided and to 1 a left-sided bias. As mentioned above, estimations of the attentional components were based on only those trials on which no events had been presented and on which participants did not press the space-bar.

## RESULTS

The significance criterion was set to *p* = 0.05 for the statistical analyses. Unless stated otherwise, differences between the three conditions were examined by using repeated-measures analyses of variance (ANOVAs) followed by *t*-tests for dependent samples. These *t*-tests were corrected for multiple comparisons using a modified Bonferroni adjustment following Keppel (1973). The significance criterion of *p* = 0.05 was multiplied with the quotient of the degrees of freedom of the examined effect and the number of comparisons. That is, the significance criterion was set to *p* = 0.05 × 2/3 = 0.033. Mauchly’s test was used to test the assumption of sphericity of the ANOVAs. The Greenhouse-Geisser correction was applied to non-spherical data. Effect sizes are stated as $\eta_{G}^{2}$ for ANOVAs (Bakeman, 2005) and Cohen’s *d*_z_ (e.g., Faul et al., 2007) for *t*-tests.

In Experiment A, a few trials in each condition had to be discarded from the analyses because of technical problems (the presentation of duplicate letters due to an error in the experimental program). These trials occurred at random because the letters were chosen randomly (see above). The mean percentage of discarded trials amounted to 5.36% (SD = 1.35%) in the low-salient event condition, to 5.24% (SD = 1.53%) in the high-salient event condition, and to 5.49% (SD = 0.95%) in the control condition. No trials were discarded in Experiment B.

### MONITORING PERFORMANCE

Since participants were not required to perform the event monitoring task in the control condition, monitoring performance was only analyzed for the low- and high-salient event conditions. The majority of participants did not respond to any events in the control condition. However, two participants in Experiment A each responded to one of the 36 events and one pressed the space-bar on two trials with no events in the control condition. In Experiment B, one participant responded once to an event and another once pressed the space-bar on a trial with no event in the control condition.

Because monitoring performance was modeled non-parametrically, the two event conditions were compared using the non-parametric Wilcoxon signed-rank test for which *r* is reported as effect size (e.g., Field et al., 2012). In Experiment A, the participants’ sensitivities for detecting events, as assessed by A′, were significantly lower in the low-salient event (*Mdn* = 0.85, *Min* = 0.65, *Max* = 0.92) than in the high-salient event condition (*Mdn* = 0.95, *Min* = 0.87, *Max* = 1.00), *z* = -4.76, *p* < 0.001, *r* = -0.75. This was replicated in Experiment B (low-salient event condition: *Mdn* = 0.87, *Min* = 0.75, *Max* = 0.94; high-salient event condition: *Mdn* = 0.95, *Min* = 0.90, *Max* = 0.98; *z* = -4.62, *p* < 0.001, *r* = -0.75).

The participants’ bias values for $B_{D}^{\prime \prime }$ were positive in both conditions in Experiment A. This indicates that they were biased for deciding that no event had been presented. Biases were stronger in the low-salient event (*Mdn* = 0.98, *Min* = 0.82, *Max* = 1.00) than in the high-salient event condition (*Mdn* = 0.92, *Min* = 0.39, *Max* = 0.99), *z* = 4.13, *p* < 0.001, *r* = 0.65. Again, this was replicated in Experiment B (low-salient event condition: *Mdn* = 0.98, *Min* = 0.73, *Max* = 1.00, high-salient event condition: *Mdn* = 0.94, *Min* = 0.74, *Max* = 0.99; *z* = 2.98, *p* = 0.002, *r* = 0.48).

In Experiment A, RTs for correct responses to events were numerically longer in the low-salient event (*M* = 666 ms, SD = 139) than in the high-salient event condition (*M* = 606, SD = 128) and this difference approached significance, *t*(19) = 2.00, *p* = 0.060, *d*_z_ = 0.45. In Experiment B, RTs were significantly longer in the low-salient event (*M* = 664, SD = 157) than in the high-salient event condition (*M* = 568, SD = 101), *t*(18) = 2.66, *p* = 0.016, *d*_z_ = 0.61. Previous research found RTs for categorizing stimuli to be longer when available categories were of higher similarity (Cartwright, 1941). Here, in the low-salient event condition, luminance increases of the fixation cross (i.e., events) were more similar to the fixation cross of displays without events compared to the high-salient event condition. Therefore, the RT results are in line with the lower sensitivities (A′) in the low-salient compared to the high-salient event condition.

### ERROR RATES AND ATTENTIONAL COMPONENTS

Mixed ANOVAs with Experiment as between-factor did not reveal any interactions between Experiment and any of the three conditions regarding error rates and attentional components (all *F*s < 1.71, all *p*s > 0.189).

In Experiment A, the participants’ error rates differed significantly between the three conditions, *F*(2,38) = 6.76, *p* = 0.003, $\eta_{G}^{2}$ = 0.066. Error rates were higher in the low-salient event (*M* = 0.18, SD = 0.08) than in the control condition (*M* = 0.13, SD = 0.08), *t*(19) = 4.35, *p* < 0.001, *d*_z_ = 0.97. Likewise, error rates were higher in the high-salient-event (*M* = 0.16, SD = 0.08) than in the control condition, *t*(19) = 2.52, *p* = 0.021, *d*_z_ = 0.56. No significant differences were observed between the error rates of the low- and high-salient event conditions, *t*(19) = 0.94, *p* = 0.361, *d*_z_ = 0.21. In Experiment B, differences between the participant’s error rates in the three conditions did not reach significance, *F*(1.47,26.48) = 3.14, *p* = 0.073, $\eta_{G}^{2}$ = 0.022. Nevertheless, *post hoc* analyses were conducted because this effect might be regarded as close to significance. As in Experiment A, error rates were higher in the low-salient event (*M* = 0.15, SD = 0.09) than in the control condition (*M* = 0.12, SD = 0.06), but this difference also did not reach significance, *t*(18) = 1.91, *p* = 0.073, *d*_z_ = 0.72. Different from Experiment A, error rates in the low-salient event condition were significantly higher than in the high-salient event condition (*M* = 0.12, SD = 0.08), *t*(18) = 3.13, *p* = 0.006, *d*_z_ = 0.72, whereas there were no significant differences between the high-salient event and the control condition, *t*(18) = 0.23, *p* = 0.821, *d*_z_ = 0.05. It shall be noted, that these differences between the error rates in the conditions could not have compromised the observed differences between the processing speed, *C*, in the conditions (see below), because these two sets of differences were always in the same direction rather than revealing trade-off effects.

**Table 1** provides descriptive statistics for the attentional components in the three conditions of both experiments. Results of ANOVAs of the attentional components are shown in **Table 2**. Results of the accompanying *post hoc* analyses can be found in **Table 3**.

**Table 1:** Descriptive statistics of attentional components.

	Low-salient event condition		High-salient event condition		Control condition	
	Exp. A	Exp. B	Exp. A	Exp. B	Exp. A	Exp. B
	*M* (SD)	*M* (SD)	*M* (SD)	*M* (SD)	*M* (SD)	*M* (SD)
*t*_0_	20.28 (12.60)	18.20 (7.71)	20.94 (12.88)	18.28 (8.44)	22.67 (13.38)	19.05 (9.89)
*K*	2.76 (0.76)	3.03 (0.88)	2.75 (0.71)	2.92 (0.74)	2.81 (0.70)	3.00 (0.72)
*C*	46.64 (15.20)	56.71 (21.66)	56.31 (24.20)	69.62 (28.97)	72.33 (24.80)	85.07 (29.50)
α	0.66 (0.76)	0.50 (0.36)	0.54 (0.34)	0.54 (0.30)	0.49 (0.41)	0.49 (0.25)
*w*_index_	0.55 (0.14)	0.51 (0.13)	0.54 (0.14)	0.48 (0.11)	0.51 (0.11)	0.47 (0.11)

*Means (*M*) and standard deviations (SD) of attentional components in the three conditions of both experiments. Described are the threshold of conscious perception, *t*_0_ (ms), the capacity of visual short-term memory, *K* (number of letters), the processing speed, *C* (letters/s), the top-down controlled attentional selectivity, α (0 indicates perfect selection, 1 indicates non-selectivity), and the laterality of attentional weighting, *w*_index_ (a value near 0 indicates a bias to the right, a value near 1 indicates a bias to the left visual hemifield).*

**Table 2:** Analyses of variance of the attentional components for the three conditions.

	Experiment A			Experiment B		
	*F* (df_n_, df_d_)	*p*	$\eta_{G}^{2}$	*F* (df_n_, df_d_)	*p*	$\eta_{G}^{2}$
*t*_0_	0.52 (1.49, 28.26)	0.546	0.006	0.17 (2, 36)	0.846	0.002
*K*	0.34 (1.49, 28.38)	0.652	0.001	1.27 (2, 36)	0.294	0.003
*C*	16.46 (2, 38)	<0.001	0.199	16.24 (2, 36)	<0.001	0.163
α	1.02 (1.14, 21.70)	0.336	0.018	0.39 (2, 36)	0.677	0.005
*w*_index_	8.48 (2, 38)	<0.001	0.024	3.63 (2, 36)	0.037	0.026

*Repeated-measures analyses of variance of the following attentional components of both experiments: the threshold of conscious perception, t0; the capacity of visual short-term memory, K; the processing speed, C; the top-down controlled attentional selectivity, α and the laterality of attentional weighting, windex. Stated are F values, degress of freedom for their numerator (dfn) and for their denominator (dfd ), p values, and $\eta_{G}^{2}$ as effect size.*

**Table 3:** Pairwise comparisons between attentional components of the three conditions.

	Low-salient event vs. control			High-salient event vs. control			Low vs. high-salient event		
	*t*(df)	*p*	*d*_z_	*t*(df)	*p*	*d*_z_	*t*(df)	*p*	*d*_z_
**Experiment A**
*C*	-5.22 (19)	<0.001*	-1.17	-3.35 (19)	0.003*	-0.75	-2.55 (19)	0.019*	-0.57
*w*_index_	3.95 (19)	<0.001*	0.88	3.00 (19)	0.007*	0.67	0.90 (19)	0.379	0.20
**Experiment B**
*C*	-5.81 (18)	<0.001*	-1.33	-2.93 (18)	0.009*	-0.67	-2.70 (18)	0.015*	-0.62
*w*_index_	2.13 (18)	0.048	0.49	0.87 (18)	0.394	0.20	2.28 (18)	0.035	0.52

Post hoc tests for Analyses of variance (**Table 1**) with significant effects. Analyzed were the processing speed, C (letters/s) and the laterality of attentional weighting, w_index_. Stated are t values with degrees of freedom (df), p values, and Cohen’s d_z_ as effect size. Comparisons were conducted with t-tests for dependent samples which were corrected for multiple comparisons using a modified Bonferroni adjustment (Keppel, 1973). *p < 0.033 (adjusted significance criterion).

The thresholds of conscious perception, *t*_0_, did not significantly differ between the conditions of the two experiments.

Likewise, VSTM capacity, *K*, did not significantly differ between the conditions of the two experiments.

In contrast, visual processing speed, *C*, differed between the conditions in both experiments. In Experiment A, participants on average processed 25.70 letter/s less in the low-salient event than in the control condition. This was replicated in Experiment B, where participants processed on average 28.35 letter/s less in the low-salient event than in the control condition. They processed on average 16.02 letters/s less in the high-salient event compared to the control condition in Experiment A. Consistently, Experiment B yielded a difference in processing speed of 15.44 letter/s. In addition, participants processed on average 9.68 letters/s less in the low-salient event than in the high-salient event condition in Experiment A. This was again replicated in Experiment B, where they processed on average 12.91 letter/s less in the low-salient event compared to the high-salient event condition.

In both experiments, there were no significant differences between the top-down controlled attentional selectivity, α, in the three conditions.

Moreover, differences between the laterality of attentional weighting, *w*_index_, were observed in both experiments. In Experiment A, values for *w*_index_ were significantly higher in the low-salient event compared to the control condition, indicating a spatial attentional bias to the left visual hemifield in the former relative to the latter condition. Likewise, values for *w*_index_ were significantly higher in the high-salient event than in the control condition. There were no significant differences regarding the values for *w*_index_ between the low- and high-salient event conditions. In Experiment B, values for *w*_index_ were also higher in the low-salient event compared to the control condition, but this difference did not reach the adjusted significance criterion. In contrast to Experiment A, however, the high-salient event and the control condition did not differ significantly regarding *w*_index_. Moreover and again different from Experiment A, values for *w*_index_ were higher in the low-salient event compared to the high-salient event condition, although this difference only approached the adjusted significance criterion. To follow-up on these results, the data from both experiments were collapsed. For the collapsed data, *w*_index_ differed between conditions, *F*(2,76) = 9.94, *p* < 0.001, $\eta_{G}^{2}$ = 0.023. Values for *w*_index_ were significantly higher in the low-salient event (*M* = 0.54, SD = 0.13) than in the control (*M* = 0.49, SD = 0.11),*t*(38) = 3.88, *p* < 0.001, *d*_z_ = 0.62, and the high-salient event condition (*M* = 0.51, SD = 0.13), *t*(38) = 2.29, *p* = 0.028, *d*_z_ = 0.37. In addition, values were significantly higher in the high-salient event condition compared to the control condition, *t*(38) = 2.55, *p* < 0.015, *d*_z_ = 0.41.

## DISCUSSION

In two experiments with identical conditions, we asked whether monitoring affects distinct TVA-based components of visual attention that were estimated from performance in a concurrent task (see Bundesen, 1990; Vangkilde et al., 2011). More specifically, monitoring of a specific object in the environment for events (luminance increases) was required. The key question was how such event monitoring affected attentional components in a concurrently performed letter-report task. This was investigated by comparing the attentional components when monitoring for events was required (low-salient event condition) and when it was not required (control condition). We were furthermore interested in whether the effects of such monitoring vary with the expected degree of perceptual salience of the event. To address this, a third condition was included (high-salient event condition), in which monitoring was required and events were of higher salience as compared to the low-salient event condition. In both experiments, events were more frequently detected (and faster responded to) in the high-salient event than in the low-salient event condition, implying that events in the former were in fact more salient than in the latter. Also, participants showed a greater bias for reporting the presence of an event when it was more salient.

The results show differential effects of event monitoring on the five TVA-based attentional components.

First, in both experiments, the temporal thresholds of conscious perception in the letter-report task did not vary depending on whether monitoring was required or not, or on whether events were of a higher salience. Monitoring did thus not affect the time necessary for visual processing to start in the letter-report task, or to initiate the race to VSTM, respectively (cf. Bundesen, 1990).

Second, storage capacity of VSTM was approximately equal in all conditions, in each of the two experiments. It hence appears as if the number of VSTM slots (cf. Bundesen, 1990; Luck and Vogel, 1997) that were available to the letters of the letter-report task did not depend on whether monitoring was necessary or not, or on whether events were more salient. In the present study, monitoring required an attentional set that defined the fixation cross as relevant source of events. A previous study suggests that sustaining an attentional set to perform a visual monitoring task and maintaining information in VSTM can interfere in some circumstances (Helton and Russell, 2013). This is compatible with the idea that activation-based maintenance in VSTM relies on neuronal resources necessary for encoding new visual information (Petersen et al., 2012; Schneider, 2013). The present results do not indicate such interference. Crucially, the monitored object, the fixation cross, did not change across experimental trials in both monitoring conditions. Based on previous evidence (for a review, see Woodman et al., 2013), one could assume that therefore the attentional set used to monitor the object was retained in long-term memory and not in VSTM. This should have decreased the costs imposed on VSTM (Woodman et al., 2013). This attentional set was only necessary in the two conditions that required monitoring but not in the control condition. Since VSTM capacity was comparable in all three conditions, it seems unlikely that the attentional set constantly occupied a slot in VSTM.

Third, strong reductions in the processing speed of the letters of the letter-report task were observed when the fixation cross was monitored compared to when it was not monitored for events. This result was obtained in both experiments, and irrespective of whether events were more or less salient. Monitoring thus decreased the portion of overall visual processing capacity that could be used for processing the letters. The idea that attentional monitoring processes call for processing resources that are needed by concurrent, ongoing tasks was also put forward in the literature on event-based prospective memory (Smith, 2003). In this research field, however, theories mainly focused on how intentions are retrieved from memory under certain trigger-conditions (McDaniel and Einstein, 2000; Smith, 2003; Einstein and McDaniel, 2005). Therefore, they did not attempt to specify the mechanisms that may underlie monitoring the environment for events. For the visual domain, this might be done using TVA (Bundesen, 1990). For the present application, the effects on the processing speed can be explained by TVA’s filtering mechanism. When the fixation cross was monitored for events, it was more relevant to the task and hence received a higher attentional weight compared to when monitoring was unnecessary. From the viewpoint of TVA’s neural interpretation, NTVA (Bundesen et al., 2005), more neuronal processing resources should have been allocated to the fixation cross due to its higher attentional weight. Consequently, less neuronal resources should have been available to processing the letters of the letter-report task, leading to a lower processing speed. Distributing neuronal processing resources with NTVA’s filtering mechanism may thus provide a means to monitor the environment for visual events. In other words, we suggest that monitoring may be accomplished via attentional weights.

Interestingly, the letters in the letter-report task were processed faster when the events’ salience was expected to be higher rather than lower. This result was again obtained in both experiments. A recent TVA-based study revealed that salience makes an additional contribution to attentional weights, independent of the task-defined relevance of object features (Nordfang et al., 2013). In the present study, the attentional weight of the fixation cross might have been adjusted to the expected salience of the events. More salient events could have allowed participants to rely more on the events’ intrinsic ability to capture attention (e.g., Theeuwes, 2004), thereby saving visual processing resources for the letter-report task. Nevertheless, the visual processing speed in the letter-report task was still reduced when monitoring was targeted at salient events compared to when it was unnecessary. The attentional weight of the fixation cross still seemed increased as a consequence of monitoring, suggesting “monitoring via attentional weights.” In sum, monitoring for salient events imposes weaker costs on visual processing speed in a concurrent task. This is partly in line with the idea that increasing the salience of events might reduce interference between an event-based prospective memory task and an ongoing task (McDaniel and Einstein, 2000). In contrast to this account, however, capturing interference effects by an estimation of visual processing speed does not imply a unitary attentional resource (cf. Allport, 1987; Neumann, 1987). Instead, it focuses on one specific attentional resource of visual processing (cf. Bundesen, 1990).

Fourth, in neither of the two experiments was the top-down controlled attentional selectivity reliably affected by the monitoring manipulation. The up-regulation of the attentional weight of the fixation cross when it was monitored for events (evident from the effects on visual processing speed, see above), thus did not seem to affect the relation between the attentional weights of target and distractor letters within the letter-report task. In the present experiments, only one object at one location in the visual field had to be monitored for events, and it remained visible throughout the relevant periods of a trial. This might resemble situations in everyday life where it is clear which location has to be monitored to detect events of interest. A question for future research may be whether this holds also for situations of location uncertainty regarding the monitored information source. For instance, adopting a less strict selectivity within a concurrent task may be advantageous in some cases. It could facilitate the detection of events at previously unknown locations which are irrelevant to the concurrent task but which are supposed to trigger retrieval of an intention from prospective memory (cf. Vangkilde et al., 2011).

Fifth, the laterality of attentional weighting seemed affected by the monitoring manipulation. Stronger biases to the left visual hemifield were observed in case less-salient events were monitored for. In Experiment A, a left-sided bias was also observed when events were more salient compared to the condition without monitoring requirements. In Experiment B, no such bias was observed in this condition. When the data was collapsed across experiments, stronger biases to the left hemifield became apparent in the low-salient compared to the other two conditions as well as in the high-salient event compared to the control condition. Therefore, these results await further replication and investigation. Monitoring requirements might have led to an increase in the experienced task-difficulty and might have been stronger for low-salient than for high-salient events. This interpretation corresponds to the higher error rates in the two event conditions, which were known to participants because of their receiving feedback. Consequently, the participants’ level of alertness may have been higher in the two event conditions and highest in the low-salient event condition. There is some evidence that alerting participants by means of warning signals can introduce left-sided biases of attentional weighting (Matthias et al., 2010). A possible interpretation of the present results may thus be that monitoring requirements can also increase the participants’ alertness level, and thereby provoked the shifts of attentional weighting to the left hemifield.

Moreover, the finding that event monitoring affected only two out of five attentional components is not compatible with the assumption of unspecific attentional resource costs (cf. Smith, 2003; Smith and Bayen, 2004).

As mentioned in the introduction, classical studies on monitoring were part of research on vigilance (Mackworth, 1948) or sustained attention (e.g., Sarter et al., 2001). The experimental task of the present study required sustained attention in the sense of attentional sets that had to be persistently maintained across trials. These attentional sets consisted of task-based priorities for target and distractor letters of the letter-report task and for the fixation cross, which was monitored for events or not. Monitoring should have affected these attentional sets. That is, it should have affected the processing priorities (e.g., attentional weights of objects) within the task. Outside the monitoring context, however, sustained attention may also mean to engage in a cognitive task (cf. McAvinue et al., 2012; Hancock, 2013). The maintained set of processing priorities would here encompass the entire task in question, as opposed to other tasks or “absentmindedness” (cf. Manly et al., 1999). On this level of analysis, sustaining attention to a task and TVA’s attentional components (threshold of conscious perception, VSTM capacity, visual processing speed, and top-down controlled attentional selectivity; estimated from performance within this task) do not appear to be interrelated (McAvinue et al., 2012).

In summary, the present study suggests that monitoring pre-specified objects in the environment for visual events reduces the speed of visual processing in a concurrent task. In such situations, “monitoring-via-attentional weights” could take place. That is, the monitored object receives an increased attentional weight and visual processing resources are redistributed accordingly. Therefore, the amount of processing resources available for the concurrent task and the corresponding visual processing speed are reduced. Moreover, monitoring for high-salient events seems to impose weaker costs on the concurrent task than monitoring for low-salient events. By adjusting attentional weights to the expected high salience of events, that is, by relying more on the events’ stimulus-driven ability to attract attention, it seems possible to save resources for the concurrent task. There is a variety of safety-critical settings where pre-specified locations or objects have to be monitored for certain events while performing other visual tasks. Performance in such settings may benefit from applying the results of the present study. Increasing the salience of events that are to be monitored for may be used to mitigate detrimental effects of monitoring on concurrent tasks. In the example above, anesthesiologists who monitor displays of a patient’s vital signs while accomplishing other tasks may perform better in these tasks when aberrations in these displays can be expected to be highly salient.

## Conflict of Interest Statement

The authors declare that the research was conducted in the absence of any commercial or financial relationships that could be construed as a potential conflict of interest.
